# Cryoprotectant-free high-pressure cooling and dynamic nuclear polarization for more sensitive detection of hydrogen in neutron protein crystallography

**DOI:** 10.1107/S2059798318005028

**Published:** 2018-07-17

**Authors:** Ichiro Tanaka, Naoya Komatsuzaki, Wen-Xue Yue, Toshiyuki Chatake, Katsuhiro Kusaka, Nobuo Niimura, Daisuke Miura, Takahiro Iwata, Yoshiyuki Miyachi, Genki Nukazuka, Hiroki Matsuda

**Affiliations:** a College of Engineering, Ibaraki University, Hitachi, Ibaraki 316-8511, Japan; b Frontier Research Center for Applied Atomic Sciences, Ibaraki University, Tokai, Ibaraki 319-1106, Japan; c Graduate School of Science and Engineering, Ibaraki University, Hitachi, Ibaraki 316-8511, Japan; d Research Reactor Institute, Kyoto University, Kumatori, Osaka 590-0494, Japan; e Faculty of Science, Yamagata University, Yamagata, Yamagata 990-8560, Japan

**Keywords:** high-pressure cooling, dynamic nuclear polarization, neutron protein crystallography, hydrogen sensitivity

## Abstract

To improve the sensitivity of hydrogen detection using neutrons, a proton-polarization technique together with a high-pressure cooling method is necessary. The highest pressure (200 MPa) used in the experiment described here enabled relatively large protein crystals to be cooled without any cryoprotectants while retaining the protein structure, and it was confirmed that high-pressure-cooled crystals diffracted to nearly the same resolution as flash-cooled small crystals soaked with cryoprotectants. Dynamic nuclear polarization was used as a proton-polarization technique for protein crystals, and ∼300 mg polycrystalline protein doped with TEMPOL gave a maximum proton polarization of 22.3% at a temperature of 0.5 K in a 2.5 T magnetic field.

## Abstract 2

To improve the sensitivity of hydrogen detection using neutrons, high-pressure cooling of a relatively large protein single crystal and proton polarization of a protein polycrystalline state have been demonstrated as preliminary experiments.

## 1. Introduction   

It is easier to detect hydrogen in macromolecules using neutron protein crystallography (NPC) than using X-ray protein crystallography (XPC). However, it may be difficult to detect H atoms involved in the chemical reactions catalyzed by enzymes using conventional neutron methods because they have low occupancies (Niimura & Bau, 2008 ▸; Blakeley *et al.*, 2008 ▸; Blakeley, 2009 ▸; Niimura & Podjarny, 2011 ▸; Niimura *et al.*, 2016 ▸). The nuclear polarization technique has been explored over a long period of time in order to attempt to overcome technical difficulties related to neutron scattering. The dynamic nuclear polarization (DNP) method can align the direction of nuclear spins by transferring the spin polarization from electrons to the nuclei so that the neutron scattering length of each element varies (Abragam & Goldman, 1978 ▸). Application of DNP to the field of neutron protein scattering has been attempted as an alternative method to deuterium substitution (Stuhrmann *et al.*, 1986 ▸; Knop *et al.*, 1992 ▸; Zhao *et al.*, 1995 ▸; Stuhrmann, 2004 ▸). At present, with the emergence of next-generation neutron sources, DNP of hydrogen in proteins is expected to improve the sensitivity of hydrogen detection by about eight times compared with normal NPC and to reduce the background from hydrogen incoherent cross-sections (Pierce *et al.*, 2010 ▸; Niimura & Podjarny, 2011 ▸; Zhao *et al.*, 2013 ▸, 2016 ▸; Tanaka *et al.*, 2013 ▸). There have been some reports of proton polarization in neutron diffraction and scattering experiments (Zimmer *et al.*, 2016 ▸; Piegsa *et al.*, 2013 ▸). Applications to NPC, however, have not yet been performed. Several technical difficulties need to be overcome in order to realize the DNP method in NPC: cooling a large protein single crystal and obtaining a higher proton-polarization rate for a protein sample doped with a suitable concentration of a radical molecule at low temperature (∼1 K) in a high magnetic field (∼3 T).

Even if DNP can be applied, a relatively large protein single crystal would still be necessary to obtain good statistics and better resolution in NPC because of the low flux of neutrons. Here, this is defined as a large crystal with each edge longer than 0.5 mm. High-pressure cooling is one of the most reliable and best methods for cooling larger crystals. In order to cool protein single crystals, a flash-cooling method with suitable cryoprotectants is generally used. When cooling, the smaller a single crystal is the better it can be cooled, because the temperature gradient inside the crystal and/or the difference in the density of ice, which depends on the temperature and pressure generated by flash-cooling, may destroy the crystal or protein structure (Mishima, 1996 ▸). When the highest pressure (200 MPa) is applied to a protein crystal, the viscosity of water becomes maximum. Immediately after this, the crystal is cooled quickly (flash-cooling), depressurized and kept in liquid nitrogen. The water then becomes vitreous or an amorphous solid (not crystalline), which is a quasi-stable state (Kim *et al.*, 2005 ▸). This high-pressure cooling method does not always require cryoprotectants, which should be examined carefully so as not to affect the crystal quality or the structure itself. The application of high pressure can have any effect on the protein structure and function (Yamada *et al.*, 2015 ▸); however, diffraction measurements show that these effects are reversible and that they almost return to the normal state and show only a small deviation (Kim *et al.*, 2005 ▸). Of course, neutrons have a low energy which is insufficient to destroy the protein crystal; however, such cooling conditions may be helpful to find a protonation state for a reaction intermediate (Casadei *et al.*, 2014 ▸). Therefore, this cooling method is very important for NPC with larger crystals using DNP.

In this study, two key experiments were performed to improve the detection sensitivity of hydrogen in a protein: high-pressure cooling as a practical experiment for diffraction and DNP as a first experiment on proton polarization in a protein doped with the highly soluble radical TEMPOL.

## 2. Materials and methods   

### 2.1. High-pressure cooling and X-ray diffraction experiment   

Lysozyme (Sigma–Aldrich catalogue No. L6876) was crystallized in a similar manner to previously (Tanaka *et al.*, 2013 ▸), except that 20, 50, 100 or 200 m*M* TEMPOL (4-hydroxy-2,2,6,6-tetramethylpiperidine-1-oxyl; Sigma-Aldrich catalogue No. 176141) was added for co-crystallization using the batch method. TEMPOL has a solubility that is more than 170 times higher than that of TEMPO (2,2,6,6-tetramethylpiperidine-1-oxyl) as a stable radical to initiate nuclear polarization.

**Figure 1:**
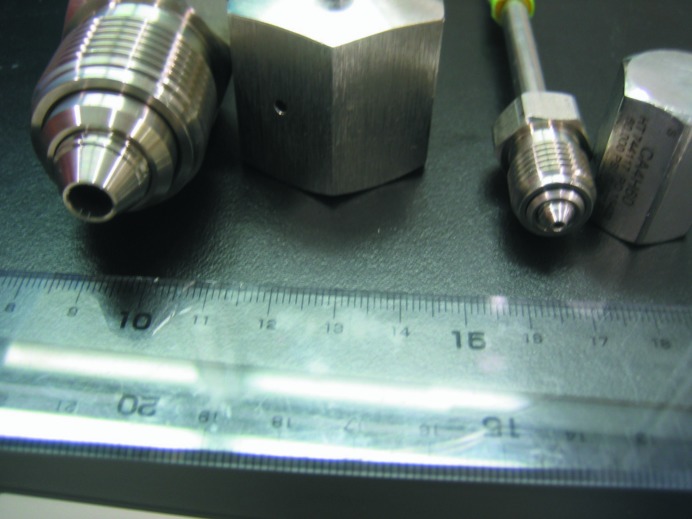
Pressure tubing for high-pressure cooling: 2.11 mm in diameter for a maximum 1 × 1 × 1 = 1 mm^3^ crystal (right) and 6.35 mm in diameter for a maximum 3 × 3 × 3 = 27 mm^3^ crystal (left).

For high-pressure cooling, a high-pressure cryocooler (HPC-201, ADC) and an EM cryopreparation chamber (CPC; Leica Microsystems) were used. The CPC is a very useful tool because it is easy to handle the cooled sample through the cooled transparent nitrogen gas under the well controlled temperature conditions and there is no need to worry about the formation of frost. As shown in Fig. 1 ▸, two kinds of pressure tubing were prepared for different sizes of crystals; the thicker one was specially ordered from ADC. It takes about 3 min for the thinner tube and about 8 min for the thicker tube to arrive at maximum pressure. No cryoprotectants were used during this time.

After cooling, the crystals were irradiated using X-ray diffractometers at synchrotron facilities under nitrogen gas at 100 K and full data were collected from each crystal. *HKL*-2000 (Otwinowski & Minor, 1997 ▸) or *XDS* (Kabsch, 2010 ▸) was used to reduce the raw data, and *PHENIX* (Adams *et al.*, 2010 ▸) and *Coot* (Emsley *et al.*, 2010 ▸) were used to analyze the structures.

### 2.2. Electron spin resonance and dynamic nuclear polarization experiment   

Electron spin resonance (ESR; JES-FA300, JEOL) measurements were made on protein single crystals doped with TEMPOL at different concentrations. One protein single crystal (0.03–0.2 mm^3^ in volume) was placed into a quartz capillary for X-rays after removing the buffer liquid on the crystal surface, and the capillary was sealed with a small amount of buffer from the crystal to prevent drying. This capillary was then placed into a normal ESR quartz capillary and ESR measurements were conducted. The number of radicals was calculated based on calibration values obtained from measurements with known TEMPOL concentrations in the same buffer solution without protein.

**Figure 2:**
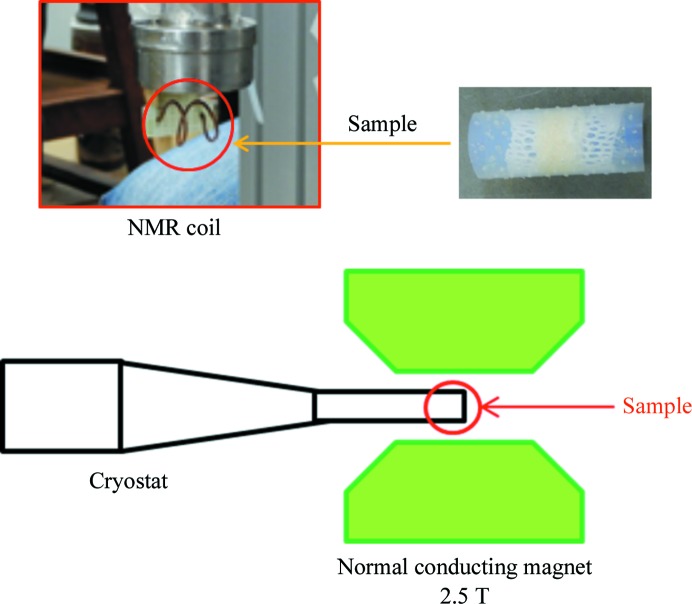
Experimental setup for DNP; the protein sample in a liquid helium-permeable container made of Teflon (top right) was placed in an NMR coil at the bottom of a cryostat (top left) and the sample in the cryostat was horizontally aligned at the centre of the 2.5 T normal conducting magnet.

To prepare the sample for DNP, about 300 mg of lysozyme polycrystals doped with 50 m*M* TEMPOL were collected after removing the moisture around the crystal by centrifuging them lightly and were used to fill a Teflon tube with a diameter of 10 mm. The small protein crystals filling the tube were sandwiched by a Teflon sheet with small holes deformed to fit the inside surface of the tube. The holes were large enough for liquid helium to permeate the sheet but were not large enough for the crystals (top right in Fig. 2 ▸). A large mass of crystal sample was necessary for the NMR coil used in this case to detect the NMR signal in the DNP experiment. The sample was then immersed in liquid nitrogen and stored until the DNP measurements. For DNP measurements, the sample in the Teflon tube was placed in the NMR coil at the bottom of a cryostat in liquid nitrogen (top left in Fig. 2 ▸). The cryostat was then moved to the centre of a magnet (bottom part of Fig. 2 ▸). In this way, the protein sample was polarized with microwaves of about 70 GHz at a temperature of 0.5 K by a ^3^He refrigerator in a 2.5 T normal conducting magnetic field.

## 3. Results and discussion   

### 3.1. High-pressure cooling of a relatively large protein crystal   

**Figure 3:**
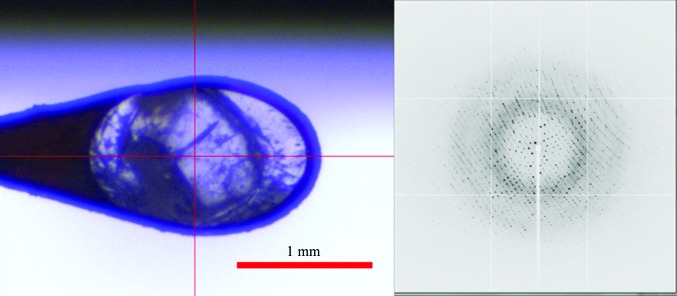
A photograph of a cooled crystal on a goniometer at the diffractometer (left) and its diffraction spots from synchrotron-radiation X-­rays (right).

A relatively large-volume lysozyme crystal (about 1 mm^3^), doped with 50 m*M* TEMPOL and with no cryoprotectants, was successfully cooled in a thick tube (6.35 mm diameter) and diffracted to a resolution of 1.34 Å on BL2S1 at the Aichi Synchrotron Radiation Center with a synchrotron source of 1.2 GeV, as shown in Fig. 3 ▸ and Table 1 ▸. A similar-sized lysozyme crystal, which was cooled in a thin tube (2.11 mm diameter) without cryoprotectants, diffracted to a resolution of 1.20 Å on BL-5A at the Photon Factory with a synchrotron source of 2.5 GeV, as shown in Table 2 ▸. On comparing these high-pressure-cooled structures with flash-cooled structures with cryoprotectants, the r.m.s.d.s of the coordinates for the main chains were found to be 0.12 and 0.17 Å for TEMPOL-doped lysozyme and TEMPOL-undoped lysozyme–sugar complexes, respectively (unpublished results). In addition, there was a case in which the overall r.m.s.d. (main chain and side chain) was 0.66 Å compared with a main-chain r.m.s.d. of 0.32 Å for ferritin (unpublished results). These r.m.s.d. values are comparable to those in previous results (Kim *et al.*, 2005 ▸).

### 3.2. ESR experiment for a protein single crystal   

**Figure 4:**
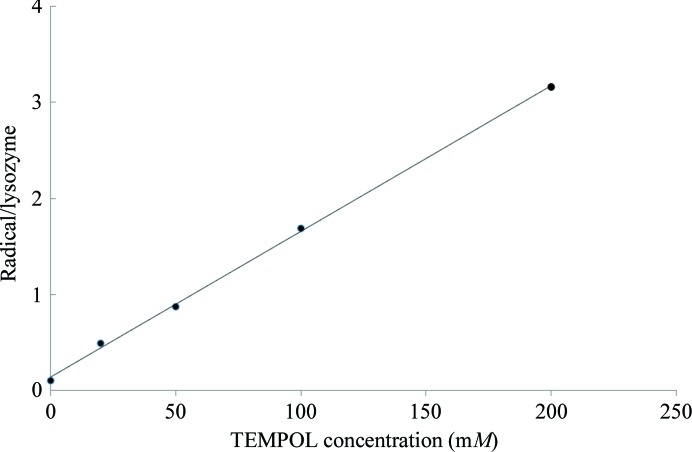
Summary of ESR measurements: dependence of the number of radicals per lysozyme protein molecule upon the TEMPOL concentration during co-crystallization with lysozyme. The straight line is the result of fitting the data.

ESR measurements were conducted for crystals grown from 0 (no TEMPOL) to 200 m*M* TEMPOL under crystallization conditions. TEMPOL has one unpaired electron (radical) per molecule. If the lysozyme sample is assumed to be crystallized in space group *P*4_3_2_1_2 and its unit-cell parameters are known, the number of lysozyme molecules can be calculated from the measured crystal volume. Moreover, from the radical numbers observed by ESR, the number of radicals per lysozyme protein molecule can be calculated for each crystal grown at a particular TEMPOL concentration. Fig. 4 ▸ shows a good linearity between the number of radicals per lysozyme protein molecule and the TEMPOL concentration in co-crystallization with lysozyme. Bunyatova (1995 ▸) and Kumada *et al.* (2012 ▸) reported that one radical per 1000 protons is suitable as a radical density for DNP. Because lysozyme has about 1000 H atoms per molecule, a 50 m*M* TEMPOL crystal was selected for the DNP experiment.

### 3.3. Dynamic nuclear polarization of the protein polycrystalline state   

**Figure 5:**
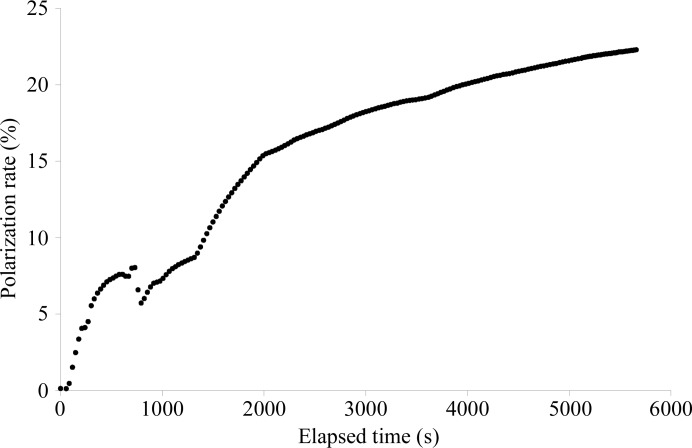
DNP measurements: the dependence of proton polarization upon the elapsed time.

After setting the temperature to 0.5 K, the magnetic field to 2.5 T and the microwave frequency to 70 GHz, it took about 90 min to obtain the maximum proton polarization rate of 22.3 ± 0.7% (Fig. 5 ▸) at a microwave power of 57 mW. This preliminary polarization experiment for the protein polycrystalline state stopped owing to a shortage of ^4^He. The polarization rate *P*_DNP_ was calculated proportionally from the area of the NMR signal *S*_DNP_ based on the thermal equilibrium polarization rate *P*_TE_ with the NMR signal area *S*_TE_, which is determined by the Boltzmann distribution for temperature *T*, *P*_TE_ = tanh[μ*B*/(*k*_B_ *T*)], as *P*_DNP_ = *P*_TE_ × (*S*_DNP_ /*S*_TE_). Here, μ is the proton magnetic moment, *B* is the magnetic field strength and *k*_B_ is the Boltzmann constant. This polarization rate is still low for the enhancement of hydrogen visibility. To increase it, the concentration of TEMPOL should be increased and/or it might be necessary to reduce the amount of dissolved oxygen in the solution around the protein in the crystals, as it has been reported that dissolved oxygen in the sample to be polarized might decrease the polarization relaxation time *T*_1_ (de Boer, 1973 ▸). In this experiment, *T*_1_ was 527 s at 1.1 K, and the temperature dependence of *T*_1_ was similar to those in other polarized samples (de Boer, 1973 ▸); thus, the sample preparation seemed to cause no problem. However, *T*_1_ should be longer to obtain a higher polarization rate. In addition, the sample-holding method should also be considered for NPC (see, for example, Zhao *et al.*, 2016 ▸).

For real DNP experiments in NPC, a measurement method to detect the polarization rate of a protein single crystal with a volume of around 0.1 mm^3^ (at a minimum) needs to be developed.

## 4. Conclusion   

As preliminary experiments for improving the sensitivity of hydrogen detection using neutrons, high-pressure cooling and proton polarization of protein crystals have successfully been demonstrated.

(i) A relatively large protein single crystal could be cooled without any cryoprotectants at 200 MPa. The crystal diffracted X-rays to high resolution and the structures were almost the same as those obtained with normal cooling according to the r.m.s.d.s.
(ii) ESR measurements on a single protein crystal showed a good proportionality between the radical concentration used in protein crystal growth and the number of radicals per protein molecule. This information is useful for finding the best concentration of radicals in a protein single crystal sample for DNP.
(iii) The first DNP experiment with TEMPOL in the protein polycrystalline state was conducted and the maximum polarization rate obtained was 22.3 ± 0.7%.

## Figures and Tables

**Table 1:** Data-collection, processing and analysis statistics for a high-pressure-cooled lysozyme crystal within thick pressure tubing Values in parentheses are for the outermost shell.

Diffraction source	BL2S1, Aichi Synchrotron Radiation Center
Wavelength (Å)	1.00
Temperature (K)	100
Space group	*P*4_3_2_1_2
*a*, *b*, *c* (Å)	78.94, 78.94, 37.64
Resolution range (Å)	1.34
Completeness (%)	99.7
*R*_merge_	0.052 (0.335)
〈*I*/σ(*I*)〉	24.4 (6.9)
*R*_work_/*R*_free_	0.2261/0.2524
No. of waters	80

**Table 2:** Data-collection, processing and analysis statistics for a high-pressure-cooled lysozyme crystal within thin pressure tubing Values in parentheses are for the outermost shell.

Diffraction source	BL-5A, Photon Factory
Wavelength (Å)	1.00
Temperature (K)	100
Space group	*P*4_3_2_1_2
*a*, *b*, *c* (Å)	78.47, 78.47, 37.01
Resolution range (Å)	1.20
Completeness (%)	94.8
*R*_merge_	0.092 (0.378)
〈*I*/σ(*I*)〉	60.0 (12.02)
*R*_work_/*R*_free_	0.213/0.237
No. of waters	153
